# User identification system based on 2D CQT spectrogram of EMG with adaptive frequency resolution adjustment

**DOI:** 10.1038/s41598-024-51791-4

**Published:** 2024-01-16

**Authors:** Jae Myung Kim, Gyuho Choi, Sungbum Pan

**Affiliations:** 1 Department of Electronics Engineering, Chosun University, Gwangju, 61452 Republic of Korea; 2 Department of Artificial Intelligence Engineering, Chosun University, Gwangju, 61452 Republic of Korea

**Keywords:** Biomarkers, Engineering

## Abstract

User identification systems based on electromyogram (EMG) signals, generated inside the body in different signal patterns and exhibiting individual characteristics based on muscle development and activity, are being actively researched. However, nonlinear and abnormal signals constrain conventional user identification using EMG signals in improving accuracy by using the 1-D feature from each time and frequency domain. Therefore, multidimensional features containing time–frequency information extracted from EMG signals have attracted much attention to improving identification accuracy. We propose a user identification system using constant Q transform (CQT) based 2D features whose time–frequency resolution is customized according to EMG signals. The proposed user identification system comprises data preprocessing, CQT-based 2D image conversion, convolutional feature extraction, and classification by convolutional neural network (CNN). The experimental results showed that the accuracy of the proposed user identification system using CQT-based 2D spectrograms was 97.5%, an improvement of 15.4% and 2.1% compared to the accuracy of 1D features and short-time Fourier transform (STFT) based user identification, respectively.

## Introduction

With the recent development of information technology (IT) and the ubiquity of Internet services in daily life, user identification methods to identify personal identity have actively been applied in electronic finance, smart medicine, access control, and healthcare services^1,2^. Conventional user identification methods that require users to have a specified password or use a specific device are prone to such problems as password loss, device loss, and theft^3^. To overcome the problems of conventional user identification methods, many researchers take advantage of the user’s unique biometric information to prove their identity. Biometric user identification technology identifies users by converting an individual’s unique physical and behavioral features into information without using a conventional password^4^.

User identification using biometric information such as face and fingerprints, which are physical features, has been applied to various fields. The problems of biometric user identification methods have attracted social attention^5^. In South Korea, user identification technology using physical feature information has been used in financial counterfeiting incidents with silicon fingerprints made by 3D printers, and user Face IDs have been recognized and disabled by the faces of the user’s family members. Overseas, a German hacker group hacked by replicating the iris with a photo of the Russian president. There was also an incident in which a cloned hand containing vein authentication information was created, and the vein authentication was deactivated by another person. Research is being conducted on using biometric signals generated inside the body to improve the security issues of user identification using biometric information^6^.

Bio-signals are electrical signals that occur inside the body and have unique characteristics of individuals. Representative bio-signals include electromyogram (EMG), electrocardiogram (ECG), and electroencephalogram (EEG)^7^. Among bio-signals, EMG signals can generate different signal patterns depending on the muscles used and the actions performed and uniform waveforms under certain conditions because the signals are acquired as the muscles move. EMG signals can be measured on the surface of the muscle, making it more convenient to measure signals compared to ECG and EEG signals. Each person has a different degree of muscle development and activity, making them unique. User identification systems based on EMG signals acquired by generating different signal patterns and unique features are being actively researched^8,9^.

EMG signals are continuous signals that generate different signal patterns owing to muscle strength over time, and user identification is being studied with features extracted from the time–frequency domain. Among the existing feature extraction methods, fast Fourier transform (FFT) can only analyze frequency components in the frequency domain, and short time Fourier transform (STFT) analyzes the time–frequency domain using a fixed window length. Thus, there is a problem in improving accuracy due to the limitation of customized time–frequency domain feature analysis in EMG signals, which are nonlinear and abnormal. Therefore, this study proposes a user identification system that solves the limitations of accuracy improvement using conventional 1D feature extraction methods in EMG signals, which are nonlinear and abnormal and improves classification accuracy by extracting constant Q transform (CQT) based 2D features whose time–frequency resolution is customized to the signal by quality factor^6^.

This study proposes a 2D CQT feature-based user identification system whose time–frequency resolution is customized for nonlinear and abnormal EMG signals. After a preprocessing step to remove unwanted noise in the EMG signal, the proposed method converts it to CQT with customized time–frequency resolution and extracts features in 2D multi-dimensions. The extracted features are converted into 2D spectrograms and then classified by convolutional neural networks (CNN) to identify the user. Via the experiments, the user identification accuracy was verified to be 97.5% when EMG signals acquired from 40 participants in the public Ninapro DB were converted into CQT-based 2D spectrograms by performing actions within the palm range. The user identification accuracy using 1D features based on 1D EMG signals was 82.1%, and the user identification accuracy using STFT-based 2D spectrograms was 95.4%. Thus, user identification accuracy improved by 15.4% and 2.1% when converted to CQT-based 2D spectrograms with customized time–frequency resolution.

## Related works

EMG signals, characterized by an individual’s unique behavioral features, are applied in various areas such as muscle activity, motion control, sign language recognition, and user identification. Early research using EMG signals was mainly conducted to detect and analyze muscle activity for medical purposes^9^. More recently, research has been conducted to identify motions using EMG signals generated by muscles. Muscle gestures can be recognized to help amputees or paralyzed patients control prosthetic hands and limbs or to identify sign language and finger spelling using EMG signals from the muscles around the forearm or wrist. Currently, research is being conducted on user identification using the characteristic that everyone shows different muscle development and activity levels^10^.

Shioji et al.^11^ built a user identification system using EMG signals. Here, EMG data acquired from the wrist was used to identify motions and users by CNN. The EMG data were acquired at a sampling rate of 128 Hz from 8 channels, and the input data was structured as $128\times 8$. The structured data was processed through a convolutional layer $3\times 3$ filter of CNN to extract features between neighboring channels and perform motion and user identification. The motion identification accuracy of the proposed method here was 94.6%, and the system was validated with 95% accuracy for user identification. Shioji et al. used a deep learning-based CNN to improve the accuracy of the user identification system.

Lee et al.^12^ proposed a user identification method using EMG signals generated when walking. Here, user identification was performed using linear discriminant analysis (LDA) based on EMG data generated by the legs when walking. The data acquired with 11 channels is used to extract the temporal features of root mean square (RMS), mean absolute value (MAV), and integrated EMG (iEMG) to identify the user. The user identification system’s accuracy was improved to 93% by extracting temporal features from the time domain of EMG signals acquired from leg muscles.

Shin et al.^13^ proposed a user identification method using a self-made EMG measurement module. Here, EMG signals were acquired using two channels, and features for user identification were extracted. Using variance (VAR), mean, zero crossing (ZC), length, and median frequency as time and frequency features, 100 features were extracted. They achieved a user identification accuracy of 95% using a self-made EMG measurement module.

Furthermore, various feature extraction methods have recently been studied to extract features from EMG signals for application in user identification. Feature extraction methods are divided into time, frequency, and time–frequency domains. Time domain feature extraction methods include MAV, slope sign change (SSC), RMS, waveform length (WL), VAR, iEMG, and ZC, and their formulas are listed in Table 1^14^.

**Table 1:** Time domain feature extraction method.

Features	Equation
MAV	$\frac{1}{N}\sum_{n=1}^{N}\left|{x}_{n}\right|$
SSC	$$\begin{array}{l}\sum_{n=2}^{N-1}\left[f\left[\left({x}_{n}-{x}_{n-1}\right)\times \left({x}_{n}-{x}_{n+1}\right)\right]\right]\\ f\left(x\right)= \left\{\begin{array}{ll}1, & \quad if \,x \ge threshold \\ 0, & \quad otherwise\end{array}\right.\end{array}$$
RMS	$\sqrt{\frac{1}{N}\sum_{n=1}^{N}{{x}^{2}}_{n}}$
WL	$\sum_{n=1}^{N-1}\left|{x}_{n+1}-{x}_{n}\right|$
VAR	$\frac{1}{N-1}\sum_{n=1}^{N}{{x}^{2}}_{n}$
iEMG	$\sum_{n=1}^{N}\left|{x}_{n}\right|$
ZC	$$\begin{array}{l}\sum_{n=1}^{N-1}\left[sgn\left({x}_{n}\times {x}_{n+1}\right)\cap \left|{x}_{n}-{x}_{n+1}\right|\ge threshold\right]\\ sgn\left(x\right)= \left\{\begin{array}{ll}1, & \quad if\;x\ge threshold\\ 0, & \quad otherwise\end{array}\right.\end{array}$$

A typical feature extraction method in the frequency domain is the FFT. The FFT converts a signal in the time domain to the frequency domain, allowing for frequency band analysis. However, owing to its temporal limitations, it cannot identify the frequency components over time^15^. A representative feature extraction method in the time–frequency domain is the STFT^16^. STFT is a method that compensates for the temporal limitations of FFT by dividing the time into short bins by setting the desired window length and Fourier transforming each bin. Thus, STFT can analyze the frequency component according to the time domain. Therefore, EMG signals whose features change over time can be analyzed as time–frequency multidimensional features by applying STFT^17^. However, the time and frequency resolutions cannot be improved simultaneously; hence, it is essential to adjust the resolution suitable for the signal by changing the parameters.

One method to simultaneously improve time–frequency resolution is the wavelet-based feature extraction approach. However, wavelet-based feature extraction methods have a drawback in that signal analysis can vary depending on the choice of mother wavelet^18^. In contrast, CQT extracts features by analyzing with a wide bandwidth at low frequencies and a narrow bandwidth at high frequencies while the bandwidth changes proportionally with the center frequency. Consequently, it improves frequency resolution in the low-frequency domain and temporal resolution in the high-frequency domain, such that the time–frequency resolution is customized to the signal by the quality factor constant^19,20^. The formulas for FFT, STFT, Continuous Wavelet Transform (CWT), and CQT are shown in Table 2.

**Table 2:** Frequency domain feature extraction method.

Features	Equation
FFT	${f}_{j}= \sum_{k=0}^{n-1}{x}_{k}{e}^{-\frac{2\pi i}{n}jk}$
STFT	$\mathrm{\rm X}\left(\mathcal{R},\upomega \right)= {\int }_{-\infty }^{\infty }x\left(\mathcalligra{t}\right)\mathcalligra{w}\left(\mathcalligra{t}-\mathcal{R}\right){\mathcalligra{e}}^{-jwt}dt=s$
CWT	${X}_{CWT}= \frac{1}{\sqrt{a}}{\int }_{-\infty }^{\infty }x\left(t\right)\psi \left(\frac{t-b}{a}\right)dt$
CQT	${X}_{CQT}\left(k, l\right)= \frac{1}{{N}_{K}}\sum_{n=0}^{{N}_{k}-1}\omega \left(n,k\right)x\left(n+lM\right){e}^{-\frac{j2\pi Qn}{N}}$

## Methods

**Figure 1:**
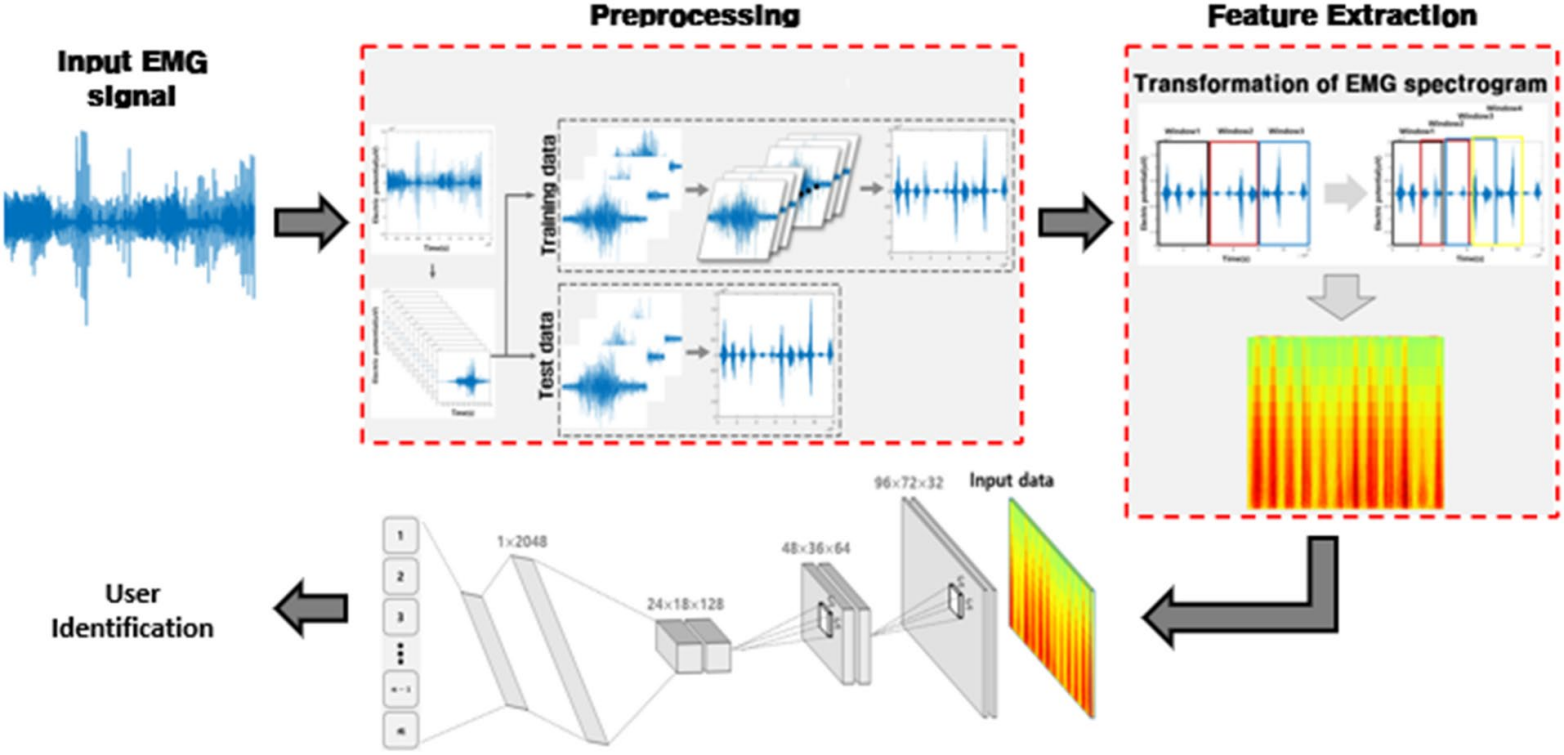
User identification system using CQT-based 2D spectrogram.

The experimental procedure of this study was approved by the Institutional Review Board (IRB) of Chosun University (IRB No. 2-1041055-AB-N-01-2023-21). The overall structure of the user identification system using CQT-based spectrograms proposed here comprises the EMG data construction process, EMG data preprocessing process, 1D EMG signal conversion to CQT-based 2D spectrograms, and final identification process, as shown in Fig. 1. EMG data is preprocessed by registering/recognizing the system and removing noise through filtering. The de-noised EMG signal is by dividing it into single-period gesture signals and combines the channels in the time domain to reconstruct the data. The preprocessed 1D EMG signal is then subjected to CQT, whose time–frequency resolution is tailored to the EMG signal, to extract multidimensional features and convert them into a 2D spectrogram. For end-user identification, a CNN is used.

**Figure 2:**
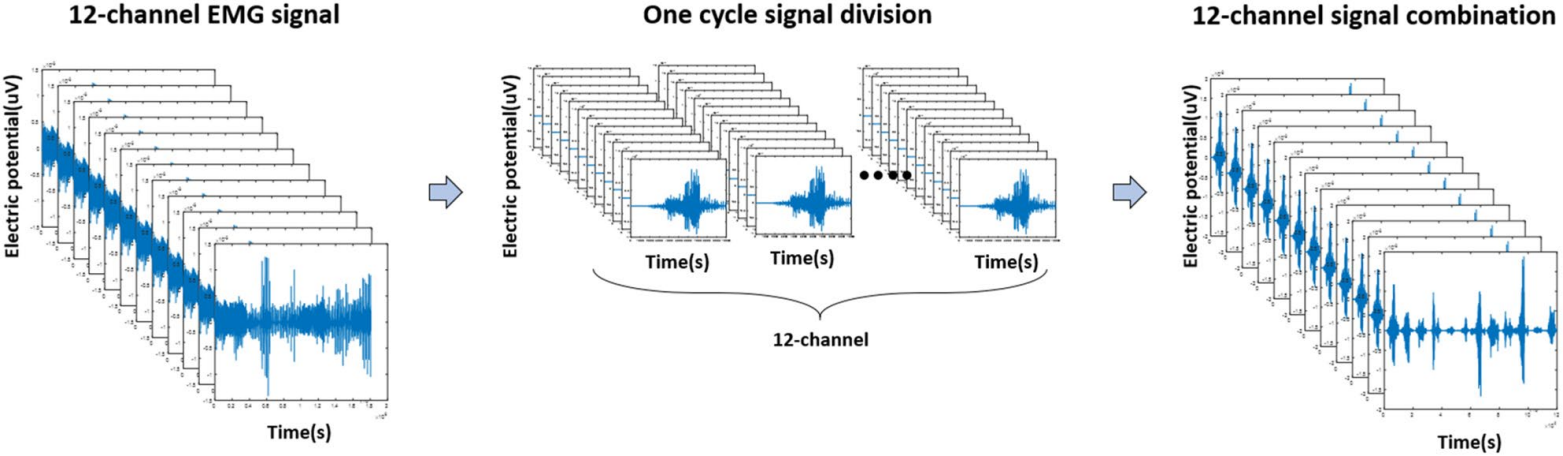
EMG signal preprocessing.

The 60 Hz power line noise in the EMG signal was removed using a notch filter, and a bandpass filter was used to extract the signal in the 10–500 Hz band containing muscle information. The de-noised EMG data was split into one cycle for each repetition of a single gesture. The EMG signal split into cycles makes it easier to see the activity of each channel. To use the information from all 12 muscles, we combine the channel-specific signals from the same gesture cycle and reconstruct them in the time domain. The process of rebuilding the EMG signal is shown in Fig. 2^6^.

CQT, a time–frequency feature extraction method, is applied to preprocessed 1D EMG signals to extract multidimensional features. FFT, a conventional frequency domain feature extraction method can only analyze frequency components and information and does not obtain time information. In addition, STFT, a method that compensates for the shortcomings of FFT, can analyze frequency components over time. However, it is limited to analyzing signals with a fixed window length in high- and low-frequency bands. Therefore, CQT, which includes time–frequency features and can analyze characteristics by frequency band, is applied to extract multidimensional features. The segmented EMG signal is converted into a 2D CQT spectrogram by the CQT equation in Table 2. In the CQT expression, $\upkappa$ is the frequency bin index; $\upiota$ is the time frame index; $\upomega$ is the window analysis of ${N}_{k}$ size, and $\mathcal{M}$ is the frameshift step. $\mathcal{Q}$ is a constant calculated as the ratio of the center frequency to the frequency band, which controls the resolution through the size of each frequency band. The bandwidth changes proportionally to the center frequency, and features are extracted by analyzing with a wide bandwidth at low frequencies and a narrow bandwidth at high frequencies.

**Figure 3:**
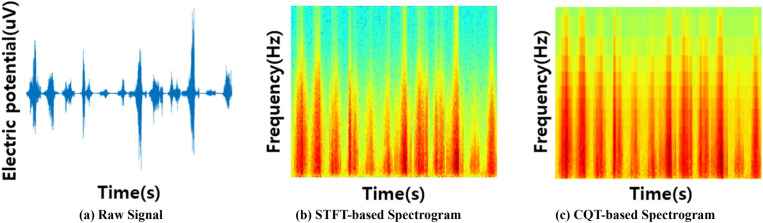
2D spectrogram conversion of EMG signals using STFT and CQT.

Because the STFT feature extraction method performs the FFT based on a fixed window length R, the resolutions of both time and frequency cannot be improved simultaneously. If $\mathcal{R}$ is small, the time resolution improves, and the frequency resolution decreases. Conversely, if $\mathcal{R}$ is large, the time resolution decreases, and the frequency resolution improves. Because the bandwidth of CQT is proportional to the center frequency, the frequency resolution is improved in the lower frequency domain, and time resolution is improved in the higher frequency domain. The feature extraction and conversion of the 2D image by applying STFT and CQT to the 12-channel combined 1D EMG signal is shown in Fig. 3. All the STFT-based spectrograms have the same resolution. However, depending on the frequency domain, the CQT-based spectrograms have a customized time–frequency resolution.

**Figure 4:**
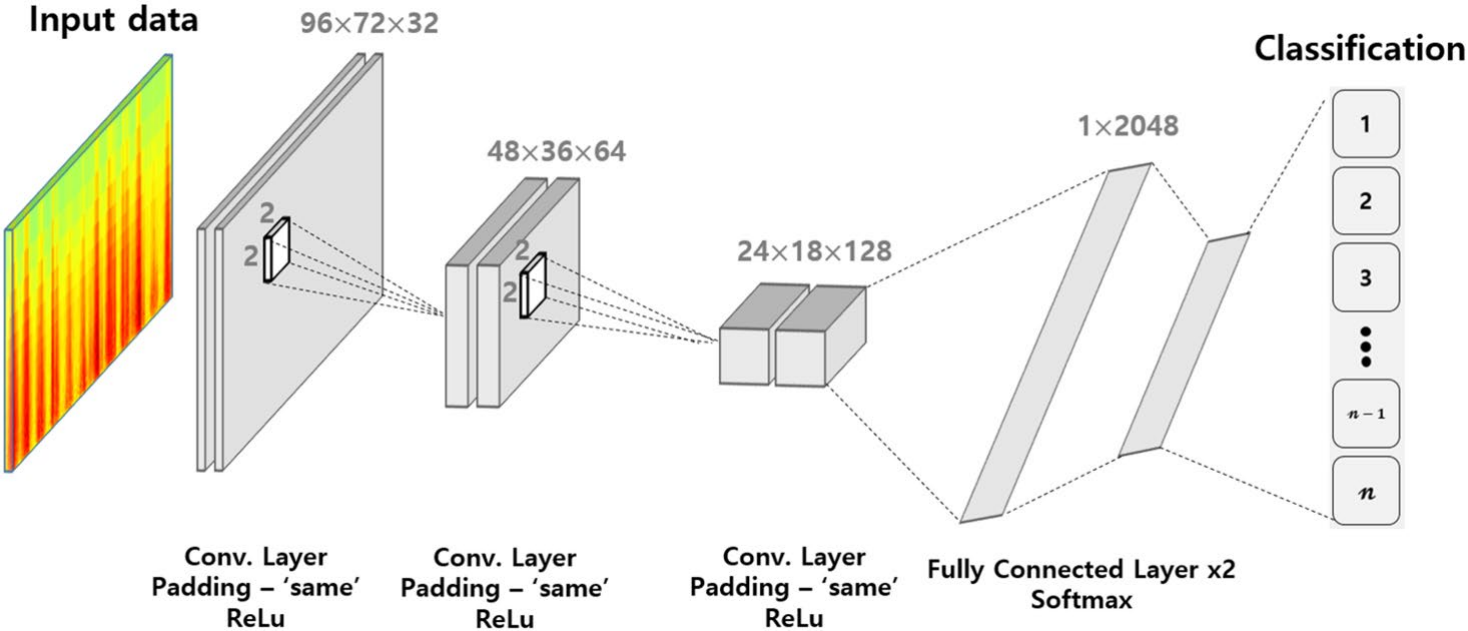
Designed CNN structure.

We used a 2D image input-based deep learning CNN for the final classification. CQT-based 2D images were used as input. The overall network structure is shown in Fig. 4. It had three convolutional layers, two max-pooling layers, and two fully connected layers, and the active function was configured with Rectified linear unit (Relu). The convolutional layer filter size was set to $3\times 3$, the pooling layer filter size was set to $2\times 2$, and the stride was set to 2. The optimization algorithm was set to adaptive momentum estimation (Adam), and the minibatch size was set to 128. The maximum number of iterations was 150, the initial weights of each layer were set randomly, and the user was identified in the output layer by Softmax^9^.

## Experimental method and results

To evaluate the performance of the user identification system using CQT-based spectrograms, we verified the accuracy by 1:N matching. EMG data were acquired from 40 participants using Ninapro DB2, and the data organization is presented in Table 3. Ninapro db2 was conducted according to the principles expressed in the Declaration of Helsinki (www.wma.net/en/20activities/10ethics/10helsinki) and it was approved by the Ethics Commission of the Canton Valais (Switzerland). Before the data acquisition began, each subject was given a thorough written and oral explanation of the experiment itself, including the associated risks; the subject would then sign an informed consent form. In total, there were 40 participants, and the adopted gestures were excluded from large gestures to identify them using hand gestures as a password^21^.

**Table 3:** Ninapro DB2 database composition.

Data	Information
Participants	40
Hand gestures (actions)	3
Channel	12
Number of repetitions (times)	6
Sampling rate (Hz)	2000

**Figure 5:**
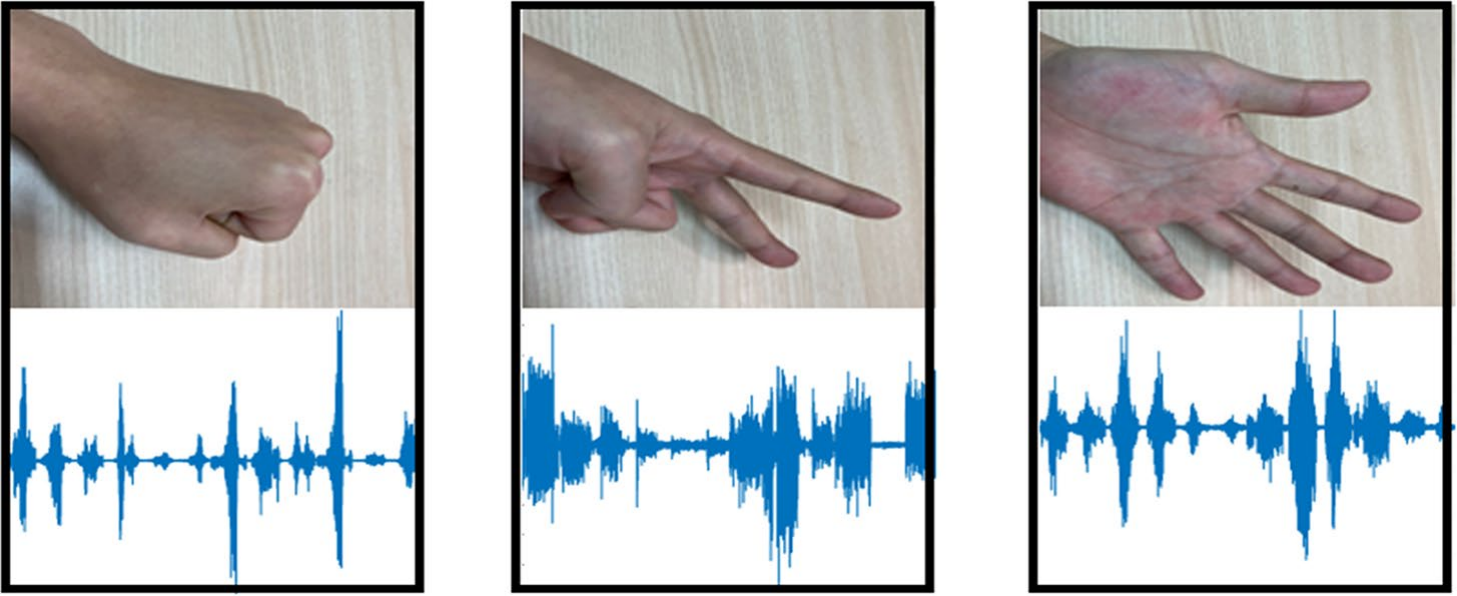
1D EMG signal according to the gesture.

Among the gestures performed within the palm, three hand gestures, rock, paper, and scissors, were used to organize the data, as shown in Fig. 5. For each participant, the three gestures were performed for six repetitions per gesture, with each gesture lasting 5 s, followed by a 3 s rest. EMG sampling was done at 2000 Hz, and 12 channels were acquired through the biceps, triceps, and brachialis muscles^22^. We used 720 data as experimental data, and the training data and test data were collected at a 7:3 ratio to analyze the accuracy of the user identification system. In addition, owing to the overfitting problem of neural networks that occurs when using small input data, this study designed and trained a network with a few layers. The accuracy was calculated by repeating the experiment a total of 5 times through k-fold cross-validation and computing the average accuracy.

**Figure 6:**
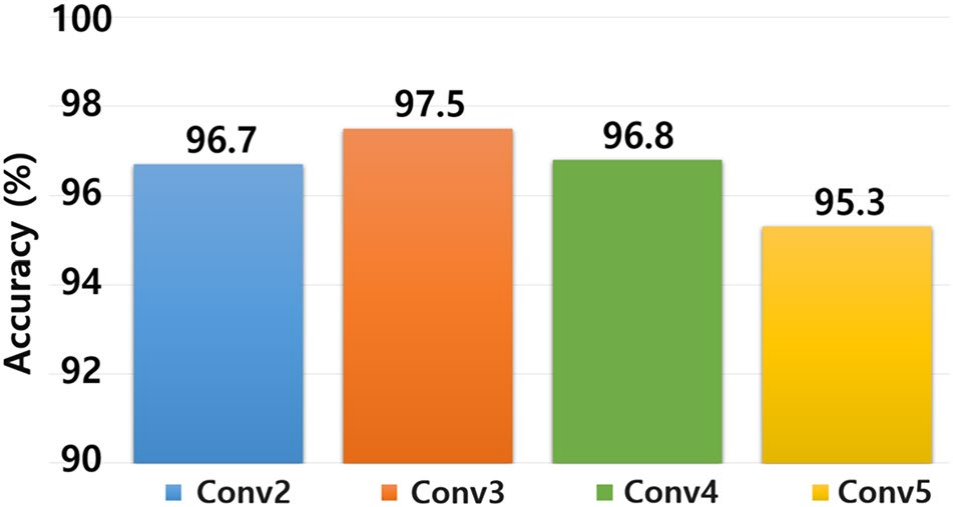
User identification performance by convolution layer.

Here, the experimental results indicated that a user identification accuracy of 97.5% was achieved when the CQT-based 2D spectrogram was applied to the proposed EMG signal. The number of bins per octave was increased to 96%. The user identification accuracy was 96.25%, with the highest accuracy when the number of bins was 12, which was set as a hyper-parameter for system validation. Furthermore, while designing the CNN, we examined the accuracy as the number of convolutional layers increased, and the user identification accuracy for each convolutional layer is shown in Fig. 6. Therefore, we adopted Conv3 for our design, which showed the highest identification accuracy, to identify users.

Table 4 compares user identification accuracy using CQT-based 2D spectrograms proposed here with popular existing methods. Shioji et al.^11^ used a CNN to extract features and perform user identification on EMG signals, which are time series data. When using the CNN designed here, like Shioji et al.’s method, we achieved an accuracy of 82.1%. Compared to feature extraction using a 1D EMG signal-based CNN, user identification accuracy improved by 15.4% when using the CQT-based 2D spectrogram proposed here. Zhai et al.^23^ also performed EMG pattern recognition by converting 1D EMG signals into STFT-based 2D spectrograms and using the spectrograms as input to a CNN. When converting EMG signals to STFT-based spectrograms as in Zhai et al. method and using the CNN designed here, we achieved an accuracy of 95.4%. The user identification accuracy was improved by 2.1% when using CQT-based 2D spectrograms compared to the STFT method using a constant window size. We utilized the wavelet-based feature extraction methods of Buelvas et al.^24^ CWT and Al Taee et al.^25^ Wavelet Scattering Transform (WST) to extract features. The extracted features were transformed into scalograms and subsequently employed with a CNN to assess user identification accuracy. The user identification accuracy using CWT and WST individually was found to be 91.7% and 96.8% respectively. Furthermore, employing CQT, which adjusts the time–frequency resolution according to the center frequency, improved user identification accuracy by 5.8% and 0.7% compared to CWT and WST, respectively. This enhancement was achieved by tailoring the time–frequency resolution based on the central frequency.

**Table 4:** Comparison of user identification accuracy among feature extraction methods.

Method	EMG signal + CNN^11^	STFT + CNN^23^	CWT + CNN^24^	WST + CNN^25^	Proposed
Accuracy (%)	82.1	95.4	91.7	96.8%	97.5
P-value	0.0301	0.0107	0.0145	0.0037	0.0092

To validate the classification accuracy results of this paper, we evaluated using precision, recall, and F1 score. The user identification accuracy based on CQT showed a performance of 97.5%. Precision was 0.9749, recall was 0.9785, and the F1 score was 0.9766, confirming the effective classification performance of the deep learning model without imbalanced or biased data sets. We also conducted Wilcoxon signed-rank tests for each set of feature data. At a 95% confidence level, the p-values were found to be less than 0.05, indicating statistically significant effects on individual identification.

The user identification method using CQT-based 2D spectrograms proposed here can be analyzed by frequency band over time. It also improves frequency resolution and temporal resolution in the low-frequency and high-frequency domains, respectively. Shioji et al. method uses a CNN to extract features from a 1D EMG signal. However, EMG signals, which are time series data measured according to behavioral features, can be difficult to identify because the features change over time. Thus, we improved the identification accuracy by extracting time–frequency multidimensional features of 1D EMG signals. In addition, the STFT method, which performs frequency analysis over time with a constant window size in the case of the Zhai et al. method, cannot simultaneously improve both time and frequency resolution. In contrast, the CQT method performs the analysis proportionately to the center frequency, enabling analysis by frequency band over time. In addition, the frequency resolution and time resolution are improved in the low-frequency and high-frequency domains, respectively. Consequently, using the CQT-based 2D spectrogram proposed here, the user identification method extracted time–frequency multidimensional features of existing EMG signals and improved user identification accuracy through time-dependent frequency band analysis. In the latest feature extraction techniques, authors Buelvas et al. and Al Taee et al. employed wavelet-based methods to classify EMG signals. While wavelet-based feature extraction methods offer the advantage of signal analysis in different frequency bands, this paper has improved user identification accuracy by extracting multi-dimensional time–frequency features from conventional EMG signals using the proposed CQT-based 2D spectrogram method. This approach enhances user identification accuracy through time-dependent frequency band analysis.

## Conclusions

Previous studies of user identification using EMG signals have used time and frequency features. EMG signals are time-series data acquired over time and generated according to each muscle’s different activation levels when performing a gesture. However, EMG signals are time-series data; thus, they cannot be repeated with constant muscle strength over time, leading to low user identification accuracy when analyzed with 1D features.

To address the limitations of accuracy improvement using 1D feature extraction methods in nonlinear and abnormal signals, we proposed a user identification system using CQT-based 2D spectrograms with customized time–frequency resolution for EMG signals. After removing the unnecessary noise in the EMG signal, features were extracted using CQT, which has time–frequency features and can be analyzed according to frequency bands. The extracted features were converted into 2D spectrograms and finally identified using CNN.

The experimental results showed that the user identification accuracy was 97.5% when the EMG signals acquired from 40 subjects gestures performed within the palm range were converted into CQT-based 2D spectrograms. We found an accuracy improvement of 15.4% compared to the method using 1D EMG signals and 2.1% compared to using STFT-based 2D spectrograms. Thus, we found that using CQT-based 2D spectrograms with customized time–frequency resolution as features proposed here improves user identification accuracy. In the future, we plan to investigate a multi-factor, multi-bio-signals-based user identification system using multiple bio-signals.

## Data Availability

The data supporting the findings of the article are available in the Ninapro DB2 at http://ninaweb.hevs.ch/ (accessed on 1 September 2020), reference number^19^.
